# H5N1 influenza vaccine induces a less robust neutralizing antibody response than seasonal trivalent and H7N9 influenza vaccines

**DOI:** 10.1038/s41541-017-0017-5

**Published:** 2017-06-08

**Authors:** Sook-San Wong, Jennifer DeBeauchamp, Mark Zanin, Yilun Sun, Li Tang, Richard Webby

**Affiliations:** 1 Department of Infectious Diseases, St. Jude Children’s Research Hospital, Memphis, TN 38105-3678 USA; 2 Department of Biostatistics, St. Jude Children’s Research Hospital, Memphis, TN 38105-3678 USA

## Abstract

Conventional inactivated avian influenza vaccines have performed poorly in past vaccine trials, leading to the hypothesis that they are less immunogenic than seasonal influenza vaccines. We tested this hypothesis by comparing the immunogenicity of the H5N1 and H7N9 vaccines (avian influenza vaccines) to a seasonal trivalent inactivated influenza vaccine in naïve ferrets, administered with or without the adjuvants MF59 or AS03. Vaccine immunogenicity was assessed by measuring neutralizing antibody titers against hemagglutinin and neuraminidase and by hemagglutinin -specific IgG levels. Two doses of unadjuvanted vaccines induced low or no HA-specific IgG responses and hemagglutination-inhibiting titers. Adjuvanted vaccines induced comparable IgG-titers, but poorer neutralizing antibody titers for the H5 vaccine. All adjuvanted vaccines elicited detectable anti- neuraminidase -antibodies with the exception of the H5N1 vaccine, likely due to the low amounts of neuraminidase in the vaccine. Overall, the H5N1 vaccine had poorer capacity to induce neutralizing antibodies, but not HA-specific IgG, compared to H7N9 or trivalent inactivated influenza vaccine.

## Avian flu: Vaccines struggle to elicit strong immune response

Evidence shows that vaccines for avian flu provoke a poorer immune response than those for seasonal human flu. Avian influenza is an emergent disease that poses a credible threat to public health, yet vaccines to treat avian flu have not performed well in clinical trials. A team of scientists led by Richard Webby of St Jude Children’s Research Hospital, United States, investigated the reasons for this by comparing vaccine’s ability to stimulate the immune system in comparison to a vaccine to treat seasonal human flu. In contrast to previous hypotheses, Webby’s group found that only the avian H5N1 flu vaccine provoked a lesser release of neutralizing antibodies compared to the H7N9 (another avian flu) and seasonal flu vaccine, and hypothesized that differences in viral surface proteins may account for the difference. The authors hope this helps to direct future research into vaccine-induced immunity.

## Introduction

Emerging avian influenza viruses, particularly those of the H5 and H7 subtypes, pose a constant pandemic threat. As vaccination remains one of the most effective strategies in controlling influenza, considerable effort has been made developing vaccines against avian influenza viruses for pandemic preparedness. However, these vaccines have not performed well in human trials, eliciting poorer antibody responses than seasonal trivalent inactivated influenza vaccine (TIV). It is now well established that unless adjuvanted, avian influenza vaccines (AIVx) require a much larger dose of antigen than TIV to achieve comparable seroconversion rates.^1–10^ This has led to the hypothesis that influenza vaccines derived from avian influenza viruses may be inherently less immunogenic than those derived from human strains.^4, 11, 12^

In this study, we compared the inherent immunogenicity of TIVs and AIVxs by evaluating the antibody responses elicited after immunization in influenza-naïve ferrets. We chose the ferret model as pre-existing immunity to influenza, which makes this study difficult to perform in humans and ferrets are able to tolerate human doses of vaccines. We immunized groups of ferrets with the commercially prepared split-virion TIV (Fluzone, Sanofi-Pasteur) or a monovalent H5N1 or H7N9 AIVx (Sanofi-Pasteur). These AIVxs were derived and prepared in the same manner as that used in past and ongoing vaccine trials (NCT02680002).^5, 6, 13^ We included the squalene oil-in-water adjuvants MF59 (Seqirus) or AS03 (GlaxoSmithKline, GSK) into our vaccination regimen as unadjuvanted vaccines have been reported to induce poor antibody responses in ferrets. These adjuvants have been licensed for use in Europe and recently in the US for select influenza vaccines.^3, 14, 15^ They have also been tested, or are currently being tested, with H5N1 and H7N9 vaccines in past and ongoing vaccine trials (NCT02680002).^1, 3, 5, 13^

In addition to evaluating the neutralizing antibody responses to the hemagglutinin protein (HA), traditionally considered the standard measure of immunogenicity in vaccine trials, we also assessed the induction of non-neutralizing IgG and neuraminidase (NA)-inhibiting antibodies after each vaccination dose. As NA-antibodies have been shown to confer protection in the absence of HA antibodies,^16–18^ there is currently a renewed interest in assessing the role of NA antibodies as an independent correlate of protection in seasonal influenza.^4, 19^ Thus, our study provides a comparative evaluation of the antibody response profile elicited by each of the vaccines tested.

## Results

### Antibody response to HA

The hemagglutination-inhibition (HAI) assay is the standard assay used to assess the immunogenicity of influenza vaccines, and measure a subset of antibodies that bind to the globular head of HA. However, viruses can exhibit different binding sensitivities to various species of red blood cells (RBCs).^20, 21^ To ensure maximum assay sensitivity, we tested the sera samples against RBCs from the following species: chicken (binds most influenza viruses), turkey, guinea pig (preferred by viruses of mammalian origin), and horse (preferred by viruses of avian origin) (Supplemental Table 1). Rg-A/Tennessee/1-560/2009 (H1_TN), which is antigenically similar to A/California/04/2009 and A/Perth/16/2009 (H3N2) (H3_Perth) agglutinated turkey RBC better than chicken RBCs. Chicken RBCs were better or comparable to the other species’ RBCs for the remaining antigens. Based on these data, turkey RBCs were used for H3_Perth and H1_TN and chicken RBCs were used with other antigens.

**Fig. 1:**
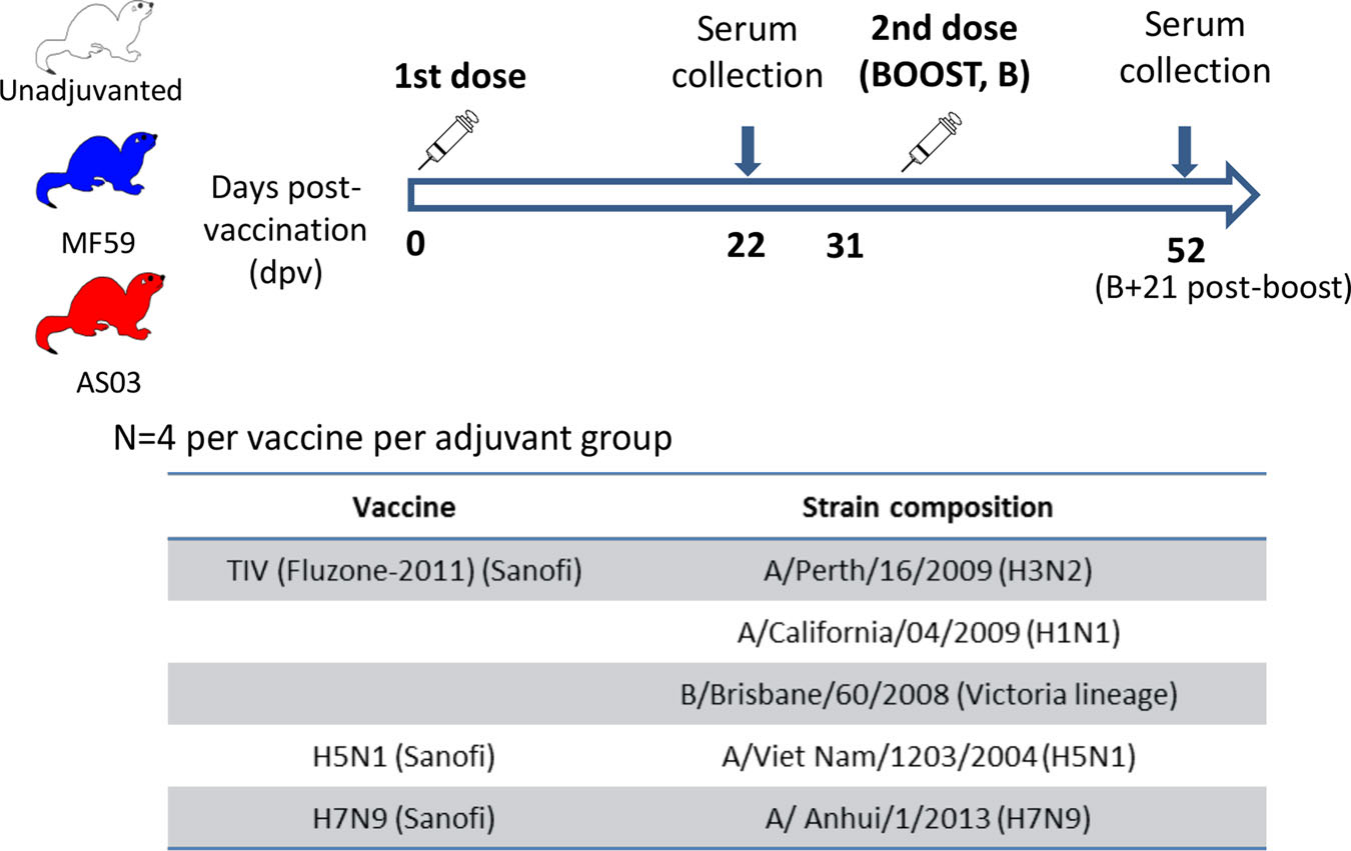
Immunization schedule and vaccine groups. Each ferret received 7.5 µg of hemagglutinin (HA) protein per vaccine strain per dose. Graphics depicted in this figure were generated in-house

**Fig. 2:**
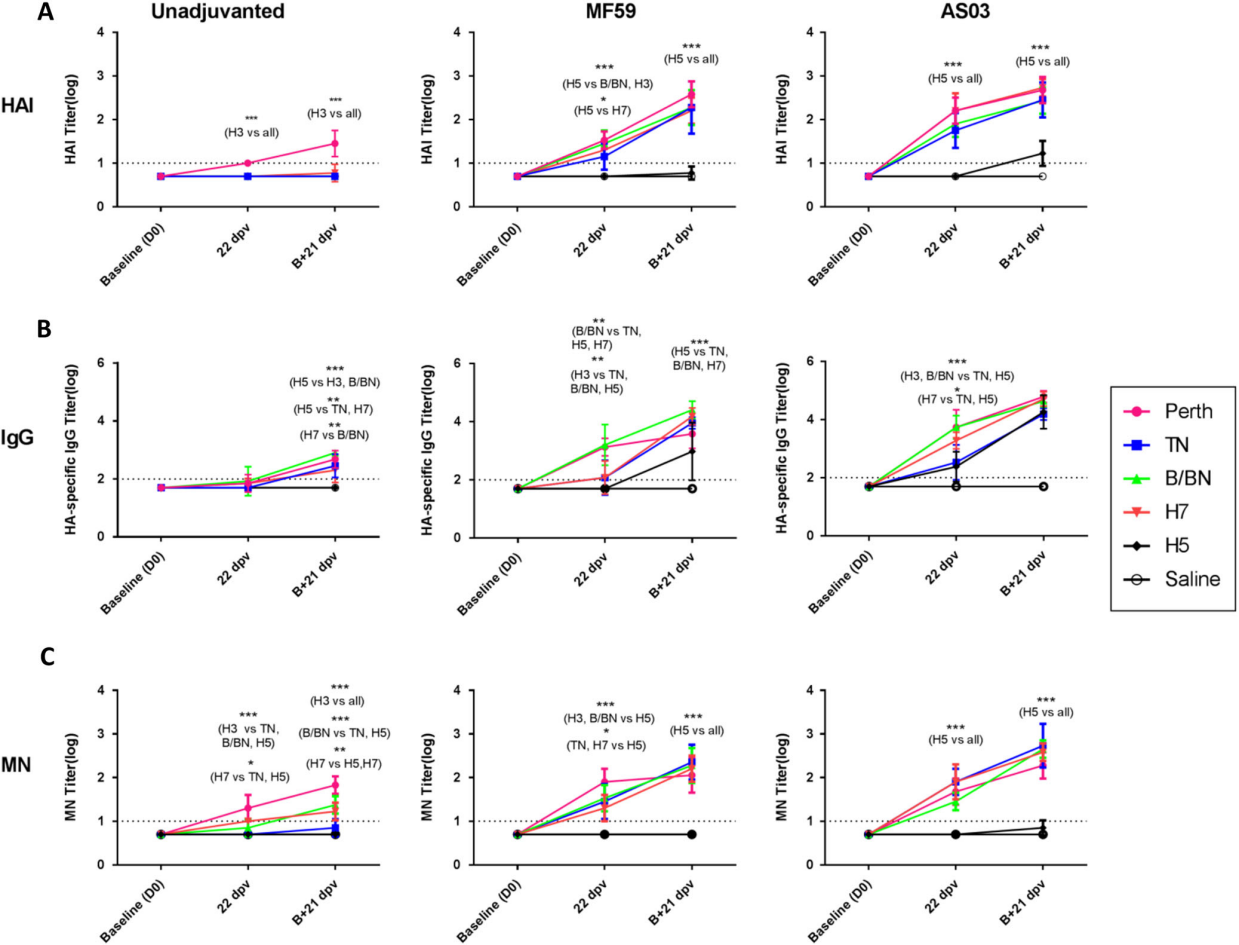
Immunogenicity profile in ferrets vaccinated with unadjuvanted, MF59 or AS03-adjuvanted TIV, H7N9 or H5N1 vaccines. Sera was collected after the first dose given 22 days post-vaccination (DPV) and the second dose (B + 21 dpv) and tested by **a** hemagglutination-inhibition (HAI), **b** ELISA for antigen-specific IgG and **c** microneutralization (MN) assay. Antibody titers are expressed as mean log_10_-antibody titer, with the *error bars* representing the standard deviation. *Dashed line* indicates the limit of assay detection; *Y* = 1 for HAI and MN assays and *Y* = 2 for ELISA. Statistically significant differences were tested by ANOVA of log-transformed antibody titers for all antigens and vaccine groups and post hoc tests with Bonferroni adjustments for multiple comparisons. *Asterisk* denotes *p*-value < 0.05, **p < 0.01, ***p < 0.001. The complete results are provided in Supplemental Fig. 1

Ferrets were divided into nine groups of four and vaccinated according to the immunization groups and schedule in Fig. 1. Summary statistics of the antibody responses measured after the first and second doses are shown in Fig. 2 (detailed antibody responses of individual ferrets are included as Supplemental Fig. 1). After two doses of unadjuvanted vaccine, HAI titers were only detected against H3_Perth (log_10_-HAI titer = 1.45 ± 0.3) (Fig. 2a), but not against any of the other viruses. This confirms previous observations that two doses of unadjuvanted vaccine (seasonal or avian) induce low levels of HAI antibodies in unprimed hosts.^22–25^

In contrast, a single dose of adjuvanted TIV or H7N9, but not the H5N1, vaccine induced detectable homologous HAI titers in the ferrets. The mean titers were relatively similar in the MF59 [(log_10_) 1.15 (H1_TN) to 1.52 (H3_Perth)] and AS03-groups [(log_10_) 1.75 (H1_TN) to 2.20 (H3_Perth)] for all antigens. Titers against H5 were undetectable for both adjuvant groups. A second adjuvanted dose boosted the titers by 0.5–1-log_10_ for all antigens (range, MF59 = log_10_ 2.2 –2.6 and AS03 = log_10_ 2.4–2.7), except H5. For the H5-group, AS03 increased the HAI titers against H5 (log_10_1.23 ± 0.29) more than MF59 (log_10_ 0.77 ± 0.15), although the mean titers still did not reach those of the other antigens.

### Total HA-specific IgG

We then evaluated whether there were differences in the induction of HA-specific IgG antibodies by enzyme-linked immunosorbent assay (ELISA). ELISA captures all antigen-binding antibodies, including those that will bind to conserved HA regions, thus increasing the sensitivity of antibody detection, but reducing its specificity.

A single dose of unadjuvanted vaccine induced no or low, HA-specific IgG titers against all antigens (Fig. 2b). After the second dose, these IgG titers increased to a greater degree in the TIV and H7N9 group (mean range, log_10_ titer = 2.45 ± 0.39 [H1_TN] to 2.90 ± 0 [B/BN]) compared to the H5, which had no detectable titer (*p* < 0.01).

After a single dose of MF59- adjuvanted or AS03-adjuvanted vaccine, IgG titers to H3_Perth and B/BN were highest (mean range, log_10_ titer = 3.1 ± 0.3 to 3.7 ± 0.6). H7 and H1_TN titers were mostly comparable in the MF59 group (log_10_ titer, 2.1 ± 0.6) and higher than the H5-group, although not significantly so. In the AS03-group, H7 titers were higher (3.3 ± 0.29) than H1_TN and H5 (log_10_ titer, H1_TN = 2.5 ± 0.6, H5 = 2.4 ± 0.6) (*p* < 0.05). The second dose of adjuvanted vaccine boosted the IgG titers in a manner that depended on the pre-existing titers. Ferrets that had higher IgG titers after the first dose generally showed a smaller increase in titer compared to ferrets with lower IgG titers post first dose. For example, titers to H3_Perth and B/BN in both adjuvant groups increased by about 0.5 to 1 log, but titers to H1_TN and H7 increased by 1.6 to 2 logs, suggesting that antibody titers had plateaued for H3_Perth and B/BN. Titers were mostly comparable across antigens in the AS03 group; but in the MF59 group, H5 antibody titers were significantly lower (*p* < 0.05).

### Microneutralization (MN) titers

We also performed MN assays using post-vaccination serum since MN assays can be more sensitive than HAI assays. Indeed, in the unadjuvanted vaccine group, MN titers were detected after the first and second doses, for H3_Perth, H7, and B/BN, but remained low or undetectable for H5 and H1_TN (Fig. 2c). In the adjuvanted vaccine groups, MN titers were detected for all antigens, except for H5 after the first dose. The second AS03-booster dose increased the MN titers only marginally for H5, despite significant increases in MN titers for all other antigens. Thus, after two doses of vaccine, MN titers generated by TIV and H7N9 were comparable and ranged from a mean of log_10_- 2.05 ± 0.65 to 2.73 ± 0.45. Titers for H5 were undetectable for MF59 or were significantly lower than those of other antigens for AS03 (*p* < 0.001). The low titers for the H5-vaccinated ferrets were further validated in an ELISA-based MN assay (Supplemental Fig. 2). Although the titers were 2-fold higher by this method, the overall titers were still significantly lower compared to other antigens (not shown).

### Antibody response to NA

To determine whether these vaccines induced any antibodies against NA and to assess if NA-antibodies contributed to the poor H5 MN titers observed, we used the enzyme-linked lectin assay (ELLA), which measures antibodies capable of inhibiting NA enzymatic function.

**Fig. 3:**
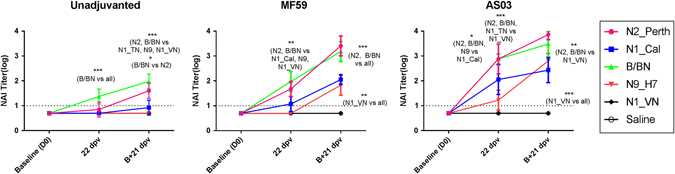
Antibody titers against NA in ferrets vaccinated with unadjuvanted, MF59 or AS03-adjuvanted TIV, H7N9 or H5N1 vaccines. Sera was collected after the first dose (22 dpv) and second dose (B + 21 dpv) and tested by enzyme-linked lectin assay (ELLA) against homologous antigens. Antibody titers are expressed as mean log_10_-antibody titer, with the error bars representing the standard deviation. *Dashed line* (at *Y* = 1) indicates the limit of assay detection. Statistically significant differences were tested by ANOVA of log-transformed antibody titers for all antigens and vaccine groups and post hoc tests with Bonferroni adjustments for multiple comparisons. *Asterisk* denotes *p*-value < 0.05, **p < 0.01, ***p < 0.001

After a two doses of unadjuvanted vaccine, NA-inhibition (NAI) titers were detected against B/BN, N2_Perth, and N1_Cal (with significantly higher titers against the former two), but remained undetectable in ferrets that received the AIVxs (Fig. 3).

After a single dose of adjuvanted-vaccines, NAI titers were highest against B/BN and N2_Perth. No or low titers were detected against N1_Cal, N1_VN and N9_H7 in the MF59 group, although N1_Cal titers were better than N9_H7 titers in the AS03 group. A second MF59- or AS03-adjuvanted dose of vaccine resulted in a robust (0.6–1.8-log_10_) increase in NAI titer against all antigens except N1_VN. The titer to N1_VN remained undetectable in the MF59 and AS03 group.

### NA content in the vaccines

**Fig. 4:**
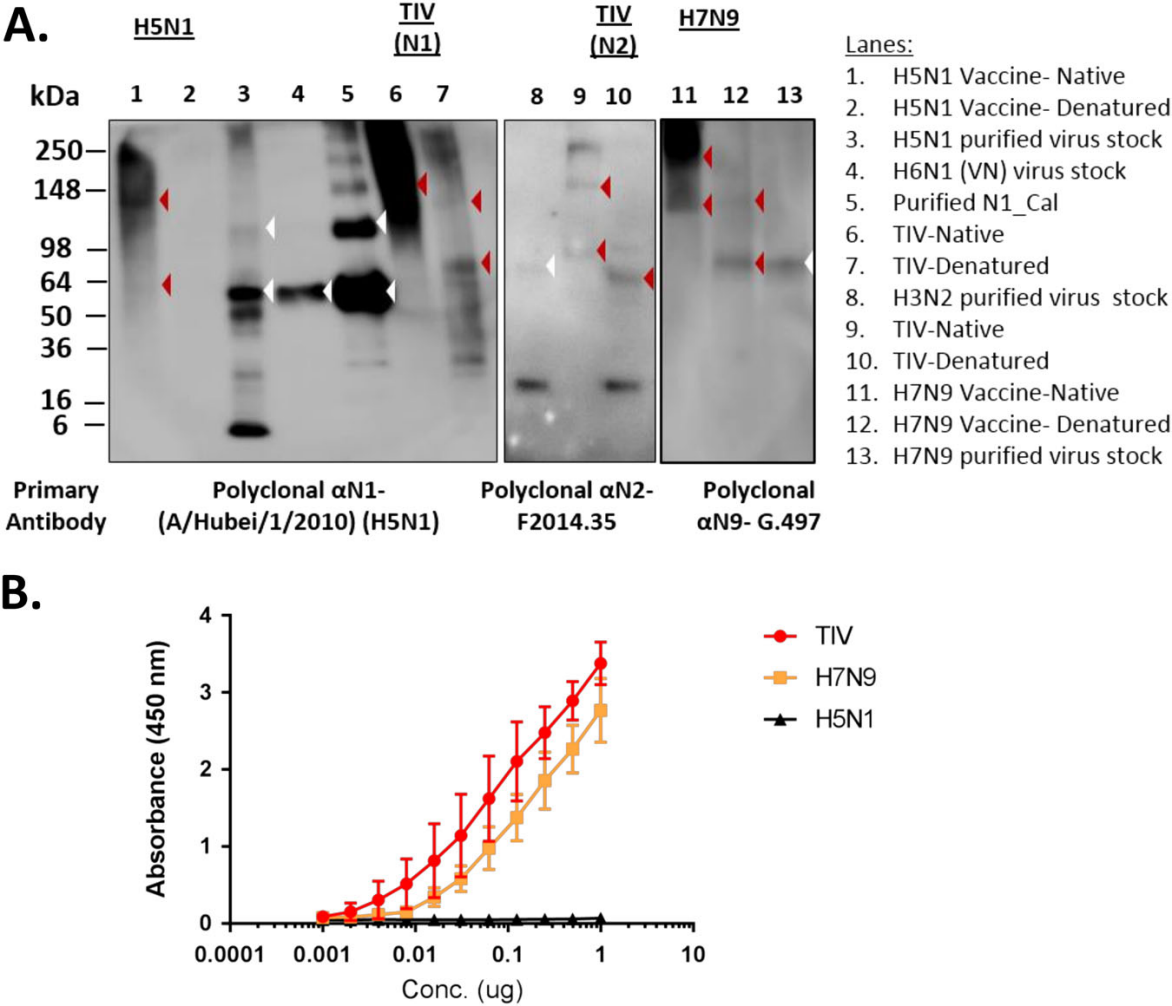
Neuraminidase (NA) contents in the H5N1, TIV and H7N9 vaccines. **a** Western blots of vaccine preparations. Native (N) and denatured (D) vaccine preparations were probed with polyclonal serum raised against the specified NA subtype. *Arrowheads* indicate the expected size of NA in the vaccines (*red*) and controls (*white*). The N1-controls (lane 3,4,5) used were a concentrated and purified reverse-genetics (rg) reassortant virus containing A/Viet Nam/1203/2004 (H5N1) HA and NA, the reassortant H6N1 (A/Viet Nam/1203/2004, NA) used in the ELLA assay, and a purified N1 protein without the ectodomain derived from A/California/04/2009 (H1N1).^16^ The N2-control used was a concentrated and purified A/Brisbane/10/2007 (H3N2) virus and the N9-control used was a concentrated and purified rg-reassortant virus containing A/Anhui/1/2013 (H7N9) HA and NA. Blots were overexposed to capture faint NA-bands in the H5N1 vaccine. Samples in lanes 1–7, 8–10 and 11–13 were processed under the same conditions respectively and run as three separate blots. No quantitative comparison is made amongst the blots. **b** Neuraminidase activity in TIV, H7N9 and H5N1 vaccines as detected by enzyme-linked-lectin assay (ELLA)

Since NA-concentration in vaccine lots is not standardized; the lack of NAI-antibody response by the H5N1 vaccine could reflect differences in the amount of NA in the vaccine preparations. To determine this, we investigated the amount and functionality of the NA-contents in each of the vaccines. In Western blots (Fig. 4a), bands corresponding to the size of NA-monomers (approximately 70 kDa for N2 and N9, and 55–60 kDa for N1) and in some cases, higher order oligomers (~140 and 250 kDa) were detected for non-denatured (native) and denatured preparations of N1 (lane 6, 7) and N2 (lane 9, 10) in the TIV and N9 in the H7N9 vaccine (lane 11, 12), confirming the presence of NA in the vaccine preparations. In the H5N1 vaccine, a faint band at approximately 55–60 kDa and a darker band at were ~ 140 kDa were detected in the non-denatured vaccine preparation (lane 1). No bands were detected in the denatured preparation (lane 2). Comparison of the N1-band intensity in the H5N1 and TIV vaccines showed that there are relatively lesser amounts of NA in the former. Due to a lack of reagents, we did not evaluate the influenza B NA content in TIV.

We further tested the integrity of the NA-components in the vaccines by ELLA. NA-activity was detected for TIV and the H7N9 vaccines, whereas no activity was detected for the H5N1 vaccine (Fig. 4b). Thus, the poor induction of NA antibodies in the H5N1 vaccine was likely due to the low amounts of intact NA protein in the vaccine preparation.^18, 26^

### Cross-reactivity of the NA-antibodies

**Fig. 5:**
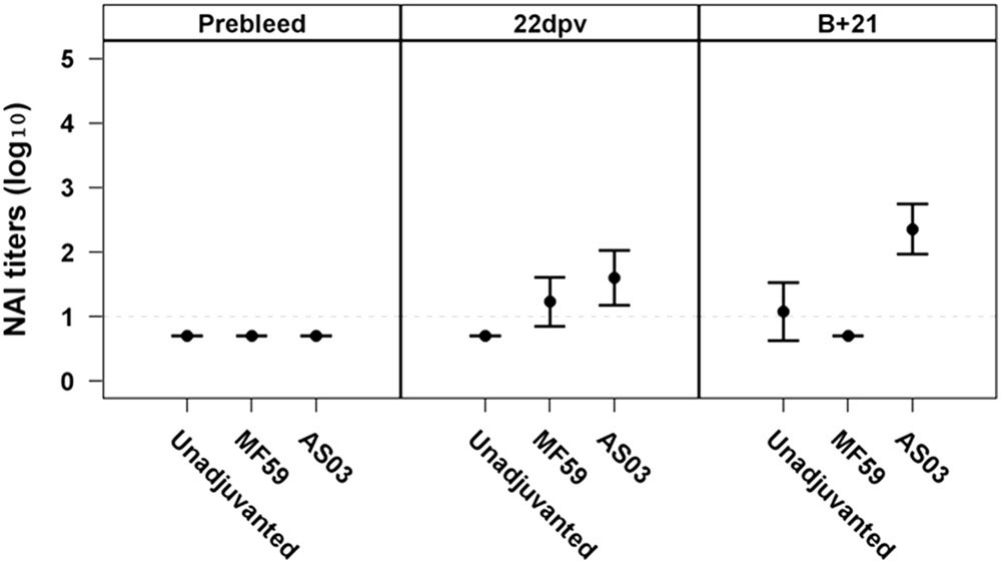
Cross-reactive antibodies against the N1 of the H5 strain elicited by TIV vaccination. Sera from the TIV vaccinated ferrets were tested for cross-reactive antibodies against N1 from the H5 virus. Antibody titers are expressed as mean log_10_-antibody titer, with the *error bars* representing the standard deviation. *Dashed line* (at *Y* = 1) indicates the limit of assay detection. Statistically significant differences were tested by ANOVA of log-transformed antibody titers for all antigens and vaccine groups and post hoc tests with Bonferroni adjustments for multiple comparisons. *Asterisk* denotes *p*-value < 0.05, **p < 0.01, ***p < 0.001

We also tested the cross-reactivity of the N1 antibodies induced by TIV against the N1 of H5. Most ferrets that received both adjuvanted-TIV vaccines had detectable cross-reactive antibodies against the N1 from H5 after the first dose (Fig. 5). However, in contrast to the AS03 group, these antibodies did not persist in the MF59 group and were undetectable by B + 21 dpv. Two ferrets that received unadjuvanted TIV also had detectable cross-reactive antibodies by B + 21 dpv. This supports previous findings that seasonal influenza vaccination or infection by H1N1 viruses may afford some pre-existing protection against H5N1 virus infections through NA-antibodies.^27–29^

### Comparison of overall immunogenicity

**Fig. 6:**
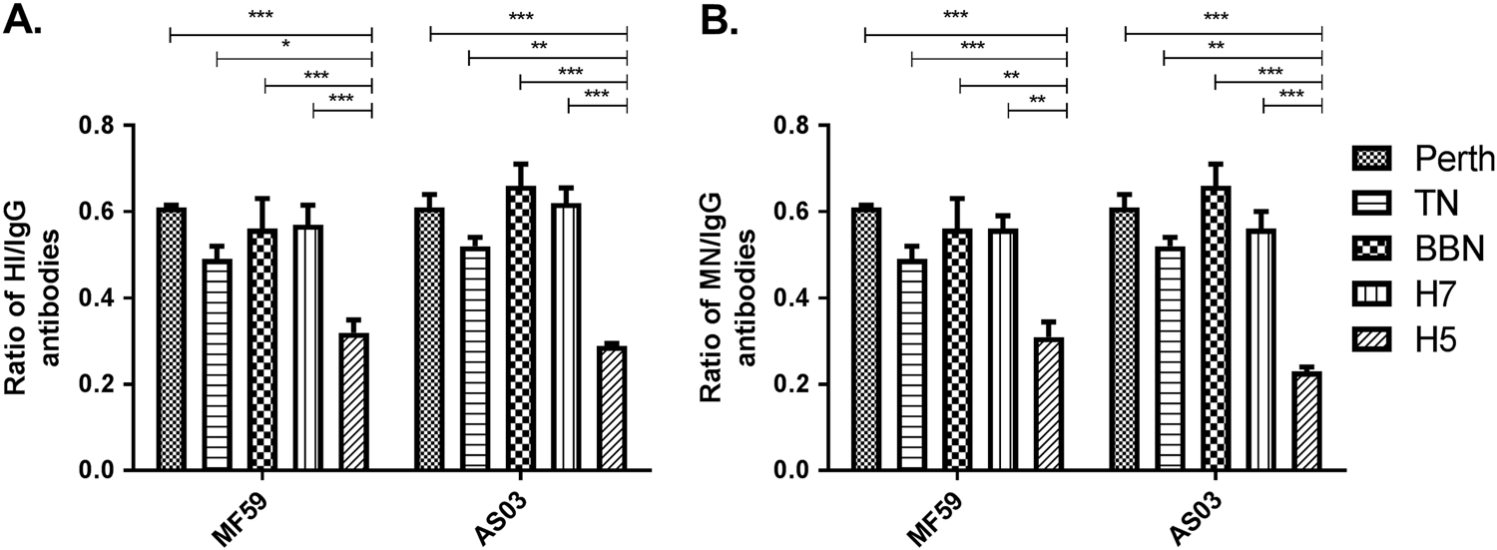
Ratio of neutralizing antibodies as measured by **a** hemagglutination-inhibition (HAI) and **b** microneutralization (MN) assays per influenza-specific IgG titer. Proportion was determined based on area-under-the-curve (AUC) of the respective antibody titer induced over time, with the assumption of a linear antibody increase between the sampling periods. Statistical significance was determined by ANOVA, with Bonferroni’s correction applied for multiple pairwise comparisons. *Asterisk* denotes *p*-value < 0.05, **p < 0.01, ***p < 0.001

To obtain a measure of the quality of the antibody response, the proportion of neutralizing antibody titer induced per total HA-specific antibody titer was assessed. To do this, we determined the area-under-the curve (AUC) for HAI, MN, and IgG antibody titers over time (with the assumption of a linear antibody increase between the first and second dose). The proportion of neutralizing antibodies was then calculated as the ratio of HAI-AUC:IgG-AUC or MN-AUC:IgG-AUC. We excluded the unadjuvanted groups from our analysis due to the lack of consistent detectable titers and compared responses from only the adjuvanted vaccine groups. Results in Fig. 6 show the mean ratio (and standard deviation) of total HAI or MN antibodies per HA-specific IgG antibody titer for each antigen in the respective vaccination group. Overall, the proportions of HAI per total antibody titers were similar to MN-per total antibody titers (ratio for HAI: 0.29–0.66 and for MN: 0.23–0.66 of the total influenza-specific IgG). Strikingly, the proportions of neutralizing H5N1 antibody titers were significantly lower in both adjuvant groups by both MN and HAI assays (range of ratio: 0.23 to 0.29) (*p* < 0.05 to *p* < 0.001) than in groups vaccinated with the other antigens (range of ratio: 0.49 to 0.66), suggesting that there may well be a difference in epitope immunodominance across the different vaccines. No consistent differences were observed between H7N9 and seasonal influenza antigens.

## Discussion

Various biological and physical factors have been explored to explain the poor immunogenicity of AIVxs reported in past vaccine trials. Our findings suggest that AIVxs were not broadly poorly immunogenic, since the H7N9 vaccine performed as well as TIV and with AS03, the H5N1 vaccine did induce comparable titers of IgG-antibodies, despite the poor neutralizing antibody response. This poor neutralizing antibody response was not due any detectable degradation of the H5N1 vaccine since the HA-epitopes were intact and appeared comparable to the other vaccines.^30^

One of the biological factors that had been reported to influence antigenicity is the glycosylation patterns of HA.^31, 32^ However, we found no obvious association between the number of potential glycosylation sites on the different HAs we tested here with the pattern of antibody responses we observed.^33, 34^ Barring these and subtype-specific differences, the other distinguishing feature between the HA of avian- and human-origin influenza viruses is the receptor-binding specificity.^35, 36^ The HA of avian viruses preferentially binds sialic acids on the cell surface with terminal α2,3 linkages, while human or mammalian influenza viruses bind sialic acids with terminal α2,6 linkages. Although this binding preference had been shown to induce a differential innate immune response in in vitro studies,^37^ how and whether this impacts the subsequent adaptive immune response after vaccination is presently unknown.

The vaccine preparations used in these trials have also been examined in vitro and found to be mostly comparable in terms of antigen presentation and HA conformation.^4, 30^ The one exception noted in both studies was that the immunogenic vaccines had more intact virus-like particles compared to poorly immunogenic vaccines. When examined under the electron microscope, the H5 vaccine (along with other poorly immunogenic H7N7 and H9N2 vaccine) lacked the large, particle-like structures that were presumably intact or split-virus particles that were in abundance in the TIV and H7N9 vaccine.^30, 38^ Lower amounts of conformationally-intact HA–NA complex in the H5 vaccine could explain the diminished HAI and NAI titers, without affecting the IgG levels. Indeed, we find that this H5N1 vaccine contained very little intact NA compared to the TIV and H7N9 vaccines. Since vaccine compositions are standardized only to HA content and contain only residual NA that may vary with the manufacturer,^39, 40^ it is unknown whether these differences in the vaccine antigen morphology were biological or due to manufacturing practices (as was discussed in ref.^ 38^). In addition, it needs to be further established whether the differences observed here for the H5N1 vaccine is strain-specific or subtype-specific.

Our findings show that the avian influenza vaccines cannot be considered to be broadly poorly immunogenic since H7N9 vaccine induced comparable antibody profile as the TIV in unprimed hosts and performed better than the H5N1 vaccines. This is in contrast to some human studies, where H5N1 vaccines appeared to be more immunogenic than H7 vaccines. This was particularly apparent at doses higher than that used in our study (i.e > 7.5 µg HA). Unadjuvanted H7 vaccination with up to 45 µg of HA, on average, appear to induce HAI titer of >40 in only 0–5% of vaccinees,^4, 5, 13^ whereas in H5N1 trials, this titer was achieved in 30–56% of vaccinees (Table 1 in ref. 38). Manufacturing practices could in part, influence a vaccine’s immunogenicity.^38^ This particular batch of H5N1 vaccine (Lot No. U10914C) appeared to be less immunogenic compared to other manufacturer’s (Table 1 in ref. 38). Results from two of the trials that used this batch of vaccine^41, 42^ and a recent H5N1 trial^3^ had a comparable range of response as the unadjuvanted H7N9 vaccine at 15 µg HA-dose.^5^ This suggests that some batches of H5N1 vaccine, particularly at lower dose may not be as immunogenic as other H5N1 vaccine preparations.

Another possible explanation for this discrepancy is that animal models still cannot recapitulate the human immune system, particularly our complex immune history. While we believe that the ferret is an appropriate model for our study objective here (comparing the vaccine’s immunogenicity), the human antibody responses to influenza can be confounded by cross-reactive preexisting immunity. It is possible, although speculative at this point, that the better responses to H5 compared to H7 vaccines in humans, is influenced by preexisting immunity, which cannot be captured by the ferret model. Indeed, studies have identified presence of some level of cross-reactive immunity, particularly within the Group 1 influenza viruses in humans.^6, 42–45^

An alternate interpretation of our findings is that the H5N1 viruses are more resistant to antibody-mediated neutralization in vitro, although to the best of our knowledge, there is no established precedence to suggest this. As we are unable to directly test this possibility, our data, when interpreted using current assay standards, suggests that the H5N1 vaccine was not as immunologically efficacious as TIV or H7N9 vaccines, even in the presence of adjuvants.

We also acknowledge that comparing the immunogenicity of TIV with monovalent H7N9 and H5N1 vaccines may potentially confound the serological data. Ideally, inclusion of the monovalent pandemic H1N1 vaccine group would have served as appropriate control. However, with careful interpretation, we think that the current experimental design and data still represents a valid comparison. Since HAI assays detect antibodies that typically show high specificity, we do not anticipate substantial impact of the multivalent formulation of TIV on HAI titers to its individual components. Impact on MN and ELISA data are a little more complex as these assays measures a broader class of antibodies. In this case, it is possible that the added antigen in the TIV could potentially have induced a higher proportion of these antibodies. However, we saw no statistically significant differences between the TIV group and the H7N9 for MN- or the IgG-titers, suggesting that the added antigens in TIV did not significantly contribute to these responses. The one exception maybe in the NAI-antibody responses as TIV appeared to perform better than H7N9, at least after the first adjuvanted dose. However, since NA-concentrations are not standardized in the vaccines, we are unsure how the multivalent formulation would affect the NAI-responses.

Finally, while there was no apparent HA-degradation detected within the limits of our assays, we considered the possibility, given the age of the H5N1 vaccine. Within this context, it would appear that vaccine degradation would compromise the ability to induce neutralizing antibodies the most, without significant impact on the raising antigen-specific IgG-titers. We have since shown that although neutralizing (HAI and MN) antibodies are the best immune correlate for protection, non-neutralizing antibodies can still reduce disease severity.^46^ Hence, despite the possibility of vaccine degradation, this batch of stockpiled vaccine can still afford protection, especially when administered with adjuvants.

Taken together, unadjuvanted vaccines are poorly immunogenic in unprimed hosts for all the influenza vaccines tested. In addition, when adjuvanted, the H7N9, but not the H5N1, vaccine were equally immunogenic as the seasonal influenza vaccines. H5N1 vaccines were particularly poor at inducing neutralizing antibodies. Thus, we propose that the poor immunogenicity observed in the H5N1 trials could possibly be due to the inability to raise HAI and NAI antibodies. While we acknowledge that measuring HA-specific IgG antibodies during AIVx vaccine trials may present a challenge due to the high immune background within the human population, we think that efforts should be made to investigate this aspect of vaccine-induced immunity.

## Methods

### Vaccine and adjuvants

The TIV was from the 2011–2012 Northern Hemisphere season (Lot No. UH442AB) and was composed of A/California/04/09 (2009 pandemic H1N1 strain), A/Perth/16/2009 (H3N2) (H3_Perth), and B/Brisbane/60/2008 (B_BN) (Victoria lineage). The H7N9 vaccine was a split-virion vaccine (Lot No. UD16397) derived from A/Shanghai/2/2013 (H7N9) virus and the H5N1 vaccine (Lot No. U10914C) was derived from a genetically modified A/Viet Nam/1203/2004 (H5N1) virus.^47^ All vaccines were manufactured by Sanofi Pasteur (Swiftwater PA). MF59 is owned by Seqirus, CSL (formerly by Novartis) (Lot No. received 091101) and AS03 by GSK (Lot No. received AA03A210A). All vaccines and adjuvants were received through the Office of Biomedical Advanced Research and Development Authority (BARDA) and tested within its suggested used-by date.

Of note, we have previously confirmed by ELISA and Western blot that the antigenic composition of these vaccines remained largely intact and comparable across the vaccines (see Fig. 3 in ref. 30).

Viruses and cells

The attenuated virus strains used in subsequent serological assays were generated using reverse genetics (rg), with the HA and NA being from the virus of interest and the six internal genes being from A/Puerto Rico/8/1934 (PR8). Rg-A/Tennessee/1-560/2009 (H1_TN) is antigenically similar to the A/California/04/2009 (H1N1) strain contained in the vaccine. Rg-H5N1 and H7N9 contained the HA and NA of A/Viet Nam/1203/2004 (with the polybasic cleavage site removed) and A/Anhui/1/2013 (H7N9), respectively. Wild-type viruses were used for A/Perth/16/2009 (H3N2) and B/Brisbane/60/2008.

Virus stocks used in this study were prepared by propagation in 10-day-old embryonated chicken eggs at 35 °C for 36 h. Aliquots were stored at −70 °C. Virus stocks were inactivated by using β-propiolactone at 0.1% (v/v) for 72 h at 4 °C.

Madin–Darby canine kidney cells used for virus neutralization assays were propagated in minimal essential medium supplemented with 10% fetal calf serum, vitamins, L-glutamine, and antibiotics at 37 °C in a humidified, 5% CO_2_ environment.

### Immunization dose and schedule

Specific-pathogen–free ferrets (4–6 months old) were purchased from Triple F Farms (Bradford County, Pennsylvania). Ferrets were divided into nine groups of four (Total = 36 ferrets, Fig. 1) and bled to collect baseline (D0) sera. Vaccine was diluted in saline to achieve the desired dose and mixed with adjuvant at a 1:1 ratio. All adjuvanted vaccine groups received the same amount of adjuvant. A control group received saline only. All ferrets received two doses of vaccine (at 7.5 µg per HA per strain per dose), given approximately 4.5 weeks apart and administered in a 0.75-mL volume by intramuscular injection. Sera collected 3 weeks post first (22 days post-vaccination (dpv)) and second dose (B + 21 dpv) were assessed for antibody responses as described below. All animal experiments were performed in accordance with guidelines approved by the Institutional Animal Care and Use Committee at St. Jude Children’s Research Hospital.

### Hemagglutination-inhibition and MN assays

Serum samples were treated with receptor-destroying enzyme (Denka Seiken, Japan), heat-inactivated at 56 °C for 30 min, and tested by hemagglutination-inhibition (HAI) assay. Sera were tested against 0.5% chicken, 0.5% turkey, 0.75% guinea pig, and 1% horse RBCs to determine the RBC species with the best sensitivity for each virus.^20^ MN assays were performed in accordance with WHO guidelines.^48^ The neutralization titer was calculated as the reciprocal dilution that inhibited virus growth as detected by the hemagglutination assay or by an ELISA-readout method.

### Enzyme-linked immunosorbent assay (ELISA)

Plates were coated with antigen at a concentration of 0.1 µg/mL at 4 °C overnight. Purified HA of each virus strain in the TIV were obtained from BEI Resources, NIAID, NIH (NR13691 for H1N1, NR42974 for H3_Perth, and NR19239 for B/BN). Baculovirus-derived recombinant HA protein from A/Viet Nam/1203/2004 (H5N1) and A/Anhui/1/2013 (H7N9) were kindly provided by Dr. Elena Govorkova and used as coating antigens for H5N1 and H7N9 vaccine group. Negative control wells were coated with buffer only. The assay was performed as previously described.^30^

### ELLA for detection of NA antibodies

The presence of NA-specific antibodies was determined via ELLA as previously described.^29^ A recombinant virus composed of the NA from A/Anhui/1/2013 (H7N9) (N9_H7), A/Viet Nam/1203/2004 (H5N1) (N1_VN), A/Perth/16/2009 (H3N2) (N2_Perth), and A/California/04/2009 (H1N1) (N1_Cal) and a mismatched HA from A/Teal/Hong Kong/W312/1997 (H6N1) was generated by using the reverse-genetics method and used as antigen. Serum samples were tested at a starting dilution of 1:10. Because no suitable antigen was available for influenza B, ELLA was performed against the whole B/Brisbane/60/2008 virus, as was previously described.^49^ For detection of NA-activity in the vaccine, 1 µg of vaccines (total for TIV) were diluted 2-fold and incubated on a fetuin-coated plate overnight.

### Sodium dodecyl sulfate polyacrylamide gel electrophoresis analysis

Vaccine (1 µg per HA strain) was mixed with Laemmli buffer (250 mM TrisHCL, 10% SDS, 30% Glycerol, 5% β-mercaptoethanol, 0.02% bromophenol blue) or non-reducing gel-loading buffer (i.e., no β-mercaptoethanol or boiling). Denatured or native vaccine samples were loaded into a mini-Protean TGX precast gel (Bio-Rad), electrophoresed for 2 h at 120 V in tris-glycine SDS running buffer (25 mM Tris, 192 mM glycine, 0.1% SDS), and then transferred to a polyvinylidene difluoride membrane via 20% methanol tris-glycine buffer (25 mM tris, 192 mM glycine, 20% methanol) for 2 h at 120 V. For N1-detection, membranes were probed with polyclonal antisera against influenza A/Hubei/1/2010 (H5N1)(Sinobiological, Cat. No. 40018-V07H) at 0.5 µg/ml. For N2-detection, membranes were probed with ferret antisera raised against N2 protein derived from A/Victoria/210/2009 (H3N2) (F2014.35) and for N9-detection, membranes were probed with goat antisera raised against N9-protein derived from A/Tern/Australia/G70C/1975 (H11N9) (G.497). As controls, purified N1 (loaded at 1 µg) from A/California/04/2009 (H1N1) were kindly provided by Dr. Rebecca DuBois,^16^ while concentrated virus stocks (loaded at 500 HA unit) and F2014.35 and G.497 were from St. Jude Children’s Research Hospital’s repository. The appropriate IgG-HRP antibodies were used as secondary detection antibody. All antibodies were diluted in 5% non-fat milk in PBST (Tween-20, 0.05%) and developed with enhanced chemiluminescent (ECL) substrate (Amersham).

### Statistical analysis

Data were analyzed by using GraphPad Prism version 5.03 and SAS 9.3. Antibody titers were log_10_-transformed, and comparison of vaccine immunogenicity was performed by using analysis of variance (ANOVA). For HAI, MN and ELLA assays, antibody titers of less than the starting dilution of 1:10 were arbitrarily set at 5, corresponding to log_10_ 0.699, while IgG-titers less than the starting dilution of 1:100 were arbitrarily set at 50, corresponding to log_10_ 1.699. Bonferroni’s corrections were made for subsequent pairwise comparisons to correct for multiple comparisons. The AUC of the antibody titers vs. time was used to calculate the proportions of HAI and MN antibodies to the total IgG antibodies of each ferret. Two-way ANOVA was used to compare AUC proportions of each antigen in both adjuvanted vaccine groups, with Bonferroni’s correction similarly applied. Mean and standard deviation are reported for continuous variables, and a *p*-value of 0.05 or less was considered to be statistically significant.

### Data availability

Antibody responses for individual ferrets are provided in Supplemental Fig. 1. All other relevant data are available from the authors.

## Electronic supplementary material

Supplemental Material
