# Supplementary material for: H5N1 influenza vaccine induces a less robust neutralizing antibody response than seasonal trivalent and H7N9 influenza vaccines
Source: NPJ Vaccines. 2017 Jun 8;2:16. doi: 10.1038/s41541-017-0017-5 (PMC5627238; doi:10.1038/s41541-017-0017-5)
Supplement: Supplementary file 1 — Supplemental Material [file 41541_2017_17_MOESM1_ESM.docx]

**Supplemental Material**

**H5N1 influenza vaccine induces a less robust neutralizing antibody response than seasonal trivalent and H7N9 influenza vaccines.**

Sook-San Wong^1^, Jennifer DeBeauchamp^1^, Mark Zanin^1^, Yilun Sun^2^, Li Tang^2^, Richard Webby^1#^.

^1^ Department of Infectious Diseases, St. Jude Children’s Research Hospital, Memphis, TN 38105-3678;

^2^ Department of Biostatistics, St. Jude Children’s Research Hospital, Memphis, TN 38105-3678

^#^Address correspondence to Richard Webby, Richard.Webby@stjude.org

**Supplemental Table 1:** The hemagglutination-inhibition (HAI) assay was tested by using red blood cells from chicken (cRBC), turkey (tRBC), guinea-pig (gpRBC), or horse (hRBC). Mean HAI titers were log_10_-transformed and standard deviations are shown in parantheses. Species that yielded the highest overall HAI titers (in bold) were used for comparisons of immunogenicity. * - p<0.05, determined by paired *t*-test against titers of cRBCs.

| **Time** | **Strain** | **0.5% cRBC** | **0.5% tRBC** | **0.75% gpRBC** | **1% hRBC** |
| --- | --- | --- | --- | --- | --- |
| 22 dpv | H3_Perth | 1.301 (0.90) | **1.577 (0.54)*** | 1.577 (0.54)* | n.d. |
|  | H1_TN | 0.699 (0) | **1.201 (0.58)*** | 1.201 (0.58)* | n.d. |
|  | B/BN | **1.351 (0.56)*** | 1.075 (0.43) | 1.276 (0.55) | n.d. |
|  | H7 | **1.401 (0.67)*** | 0.850 (0.30) | 0.699 (0) | 0.875 (0.35) |
|  | H5 | 0.699 (0) | 0.699 (0) | 0.699 (0) | 0.699 (0) |
| B+21 dpv | H3_Perth | 1.953 (1.12) | **2.355 (0.78)*** | 2.355 (0.71)* | n.d. |
|  | H1_TN | 1.853 (0.92) | **1.903 (1.0)** | 1.828 (0.96) | n.d. |
|  | B/BN | **1.803 (0.85)** | 1.577 (0.86) | 1.928 (0.93) | n.d. |
|  | H7 | **1.903 (0.88)*** | 1.602 (0.74) | 1.577 (0.74) | 1.602 (0.74) |
|  | H5 | **0.899 (0.29)*** | 0.774 (0.18) | not tested | 0.899 (0.29) |

n.d.: not done due to the lack of virus binding to hRBCs

**Supplemental Figure**

**Supplemental Figure 1:** Immunogenicity profile of individual ferrets vaccinated with unadjuvanted, MF59 or AS03-adjuvanted TIV, H7N9 or H5N1 vaccines. Sera was collected after the first dose given 22 days post-vaccination (DPV) and the second dose (B+21 dpv) and tested by (A) hemagglutination-inhibition (HAI), (B) ELISA for antigen-specific IgG (C) microneutralization (MN) and (D) neuraminidase inhibition (NAI) assay. Dashed line indicates the limit of assay detection; Y = 1 for HAI, MN and NAI assays and Y = 2 for ELISA.

**
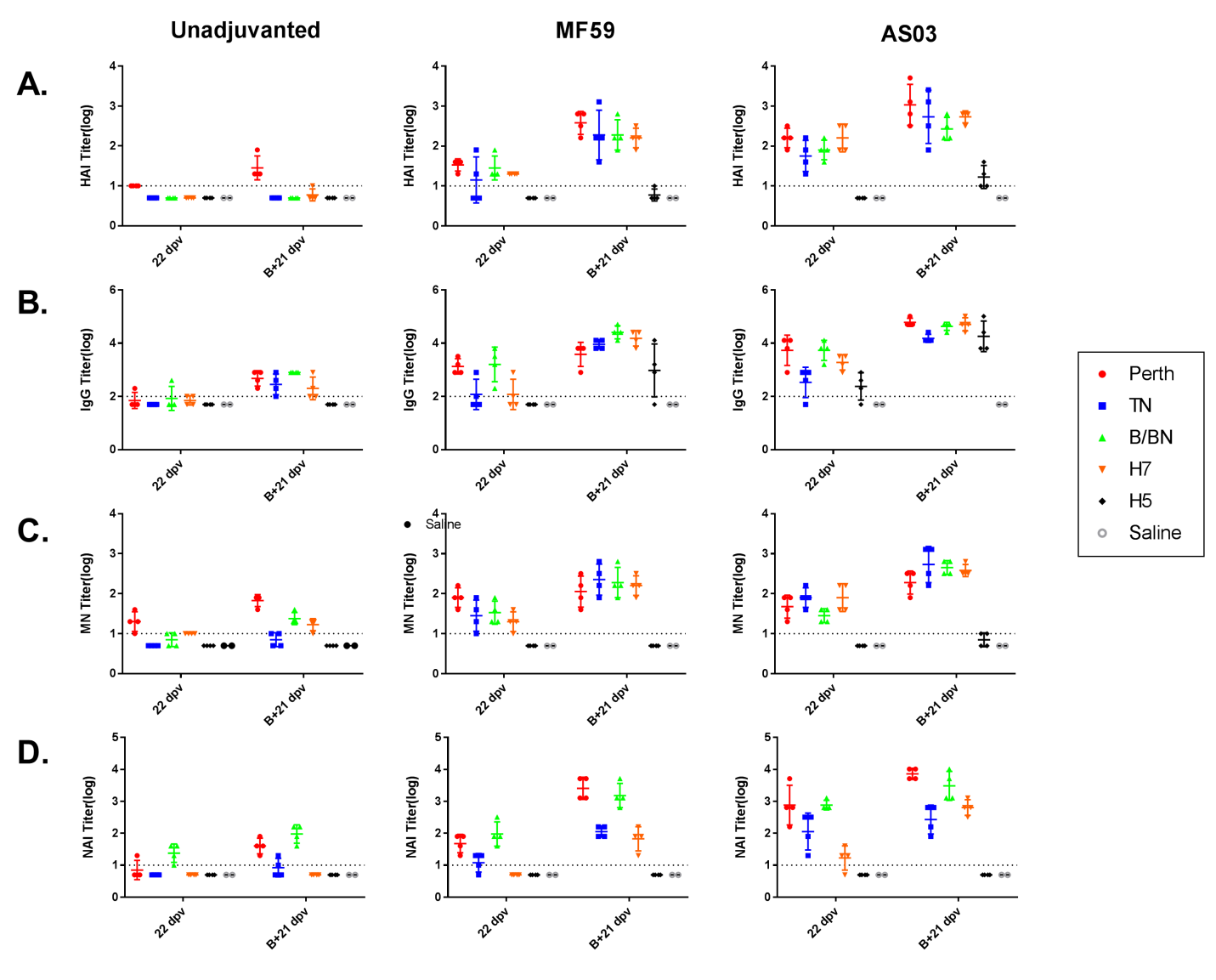
**

**Supplemental Figure 2:** Microneutralization (MN) titers for the H5N1-vaccinated ferrets when tested ELISA-based microneutralization assay. No significant differences in titers were detected between the two assays’ readout methods. Antibody titers after the first and second dose were denoted by 22 dpv and B+21 dpv respectively.


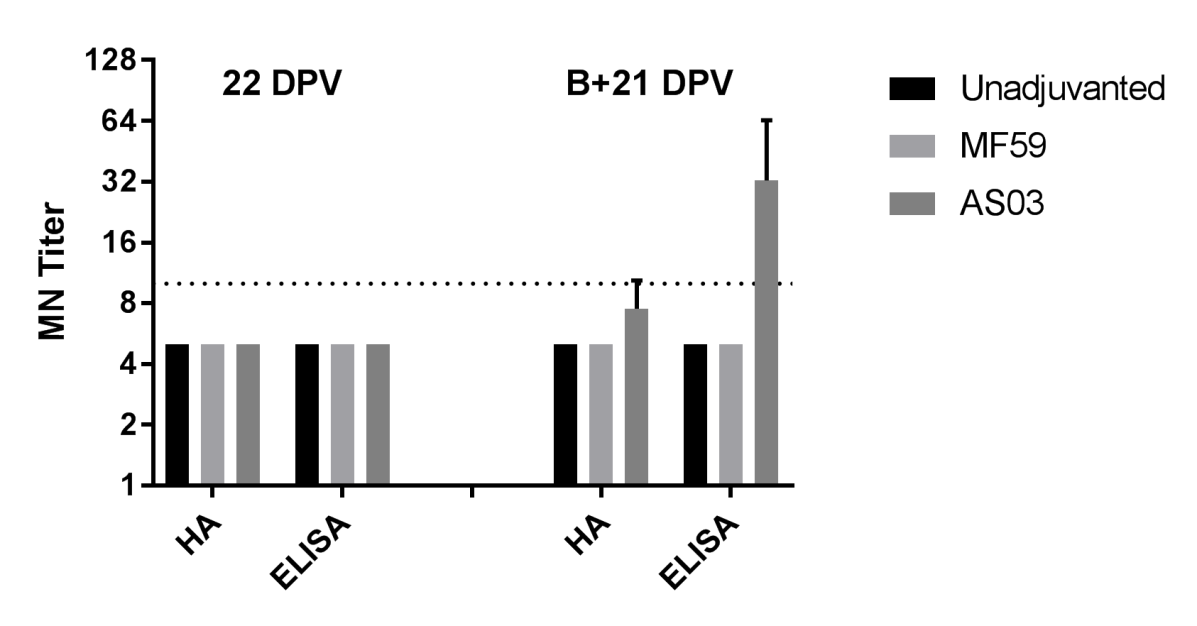


ELISA-based microneutralization assay

The assay was performed according to guidelines in the WHO Manual for Diagnosis of Influenza. The virus and sera mixture were added to plated MDCK cells in a 96-well plates as described in Materials and Methods in the main text. After 24-hours-incubation, cells were fixed in cold 80% acetone (diluted in PBS) for 20 mins. Cells were then washed three times with PBS. A mixture of goat-raised anti-NP monoclonal antibodies were added at 1:1000 dilution in 100 µl volume and incubated for 1 hour at room temperature. Cells were washed and subsequently incubated with anti-goat IgG-HRP conjugated antibodies for another hour. Cells were washed and colorimetric reaction were developed by the addition of 3,3’,5,5’-tetramethylbenzidine (TMB) (Sigma). Reaction was stopped according to the manufacturer’s instructions and the absorbance read at 450 nm. The 50% virus neutralization antibody titer was determined as the last sera dilution that gave an OD reading of less than 50% of the virus-only control wells (after subtracting the background from wells contrining uninfected cells).
